# Low-depth genotyping-by-sequencing (GBS) in a bovine population: strategies to maximize the selection of high quality genotypes and the accuracy of imputation

**DOI:** 10.1186/s12863-017-0501-y

**Published:** 2017-04-05

**Authors:** Jean-Simon Brouard, Brian Boyle, Eveline M. Ibeagha-Awemu, Nathalie Bissonnette

**Affiliations:** 1 Research and Developent Center of Sherbrooke, Agriculture and Agri-Food Canada, Sherbrooke, QC Canada; 2 Plateforme d’analyses génomiques, Institut de biologie intégrative et des systèmes, Université Laval, Quebec City, QC Canada

**Keywords:** Bovine, Imputation, Genotyping-by-sequencing, Paratuberculosis, SNP, Genome-wide association study

## Abstract

**Background:**

Genotyping-by-sequencing (GBS) has emerged as a powerful and cost-effective approach for discovering and genotyping single-nucleotide polymorphisms. The GBS technique was largely used in crop species where its low sequence coverage is not a drawback for calling genotypes because inbred lines are almost homozygous. In contrast, only a few studies used the GBS technique in animal populations (with sizeable heterozygosity rates) and many of those that have been published did not consider the quality of the genotypes produced by the bioinformatic pipelines. To improve the sequence coverage of the fragments, an alternative GBS preparation protocol that includes selective primers during the PCR amplification step has been recently proposed. In this study, we compared this modified protocol with the conventional two-enzyme GBS protocol. We also described various procedures to maximize the selection of high quality genotypes and to increase the accuracy of imputation.

**Results:**

The *in silico* digestions of the bovine genome showed that the combination of *Pst*I and *Msp*I is more suitable for sequencing bovine GBS libraries than the use of single digestions with *Pst*I or *ApeK*I. The sequencing output of the GBS libraries generated a total of 123,666 variants with the selective-primer approach and 272,103 variants with the conventional approach. Validating our data with genotypes obtained from mass spectrometry and Illumina’s bovine SNP50 array, we found that the genotypes produced by the conventional GBS method were concordant with those produced by these alternative genotyping methods, whereas the selective-primer method failed to call heterozygotes with confidence. Our results indicate that high accuracy in genotype calling (>97%) can be obtained using low read-depth thresholds (3 to 5 reads) provided that markers are simultaneously filtered for genotype quality scores. We also show that factors such as the minimum call rate and the minor allele frequency positively influence the accuracy of imputation of missing GBS data. The highest accuracies (around 85%) of imputed GBS markers were obtained with the FIMPUTE program when GBS and SNP50 array genotypes were combined (80,190 to 100,297 markers) before imputation.

**Conclusions:**

We discovered that the conventional two-enzyme GBS protocol could produce a large number of high-quality genotypes provided that appropriate filtration criteria were used. In contrast, the selective-primer approach resulted in a substantial proportion of miscalled genotypes and should be avoided for livestock genotyping studies. Overall, our study demonstrates that carefully adjusting the different filtering parameters applied to the GBS data is critical to maximize the selection of high quality genotypes and to increase the accuracy of imputation of missing data. The strategies and results presented here provide a framework to maximize the output of the GBS technique in animal populations and qualified the *PstI*/*Msp*I GBS assay as a low-cost high-density genotyping platform. The conclusions reported here regarding read-depth and genotype quality filtering could benefit many GBS applications, notably genome-wide association studies, where there is a need to increase the density of markers genotyped across the target population while preserving the quality of genotypes.

## Background

Genome-wide association studies (GWAS) are powerful tools for correlating genetic variations to a wide range of traits and diseases. The success of GWAS relies on the availability of a large number of markers, which are typically derived from commercial high-throughput technologies capable of genotyping tens or hundreds of thousands of single-nucleotide polymorphisms (SNPs). However, the per-sample cost associated with these technologies could be prohibitive when large sets of individuals are to be genotyped. To reduce the cost associated with SNP discovery and genotyping, various protocols targeting only a fraction of the genome using restriction enzymes were developed [1]. Genotyping-by-sequencing (GBS) has supplanted these methods and provides a low cost per sample, a low requirement of genomic DNA (100 ng), technical simplicity in the library preparation procedure, and compatibility with DNA barcoding [2, 3]. The use of different DNA barcoded adaptors for each library allows to pool them for sequencing (Multiplexing level up to 384). Briefly, a unique DNA barcode (4–9 bp) is ligated to the digested fragments of each library in order to associate the raw sequences to their corresponding sample. During the PCR amplification of the construction of libraries, the specificity of the forward primer prevents the amplification of fragments that would contain more than a single barcode [1]. Initially developed with one enzyme for crop plants [2], GBS has been successfully extended to a two-enzyme system by combining a ‘rare cutter’ and a ‘common cutter’ in order to achieve uniform reduction of the complexity of the genome [4]. Most GBS approaches also use methylation-sensitive restriction endonucleases to reduce the portions of repetitive DNA among the digested fragments, an advantage when working with large and complex genomes. Recently, the GBS technique was used in cattle by De Donato et al. [5] and Ibeagha-Awemu et al. [6] to identify a total of 63,697 SNPs (47 cattle animals) and 515,787 SNPs (1276 cows), respectively. Interestingly, variants detected through sequencing methods such as GBS are less prone to ascertainment bias in comparison with variants obtained from array-based genotyping platforms. For example, a large portion of the markers present on the bovine SNP50 BeadChip (Illumina) were discovered by the alignment of random shotgun reads from six breeds to the Hereford reference sequence [7] and were found to be surprisingly underrepresented in the markers derived from the whole-genome resequencing of a Holstein bull [8]. Bovine SNP50 markers also exhibit higher frequencies than random SNPs, a situation that may not facilitate the identification of complex dairy cattle traits, because many of them are suspected to rely on low-frequency (minor allele frequency [MAF] = 0.5% to 5%) and rare (MAF < 0.5%) variants [6]. Interestingly, those sequencing studies identified a large proportion of novel SNPs, suggesting that the SNP signature of a complex trait in cattle would be incomplete if a BeadChip array is used.

One of the notable features of GBS is the production of large amounts of missing data owing to the presence of variations in the restriction sites, a consequence of individual genetic divergence, differential methylation, and technical issues resulting from the library’s complexity or low sequence coverage [3]. Strategies that have proved successful for dealing with missing GBS data include adjusting the level of multiplexing, changing the choice of restriction enzyme(s), and imputing genotypes [3]. Recently, Sonah et al. [9] proposed an alternative approach to achieve a greater reduction in complexity and thereby reduce the amount of missing data produced by the GBS method. Working with eight diverse soybean lines, those authors described a modified GBS library preparation protocol that takes advantage of selective amplification during the PCR amplification step to increase the number of SNPs called and their associated sequence coverage. Exploring the extent to which missing data can be tolerated in GBS datasets, the same group also showed that SNPs can be called with high accuracy when up to 80% missing data is tolerated, given that imputation is used to fill in the missing genotypes [10].

In the present study, mass spectrometry and bovine SNP50 BeadChip arrays were used to validate bovine GBS genotypes obtained from two methods in library preparation: a typical two-enzyme method with conventional primers [4] and a method with selective-primer [9]. We discovered that the latter approach resulted in a substantial proportion of miscalled genotypes and should be avoided for livestock genotyping studies. In contrast, we found that the conventional-primer method could produce a large number of high-quality genotypes provided that appropriate filtration criteria were used. We also assessed the performance of two programs (BEAGLE and FIMPUTE) for imputing the missing genotypes and evaluating the relevance of combining SNP50 arrays and GBS datasets. We demonstrated here that a careful analysis of GBS data can produce, at relatively low cost, accurate genotypes at tens of thousands of loci in an animal population. The strategies developed in this study may facilitate the use of GBS to obtain genotype variants that are largely independent from those present on SNP arrays. In future studies, these strategies could be used at a larger scale to identify genomic regions associated with susceptibility or resistance to complex diseases such as bovine paratuberculosis.

## Methods

### Animals

A total of 48 dairy cows from various farms in the province of Quebec, Canada, were selected on the basis of the each animal’s infection status for bovine paratuberculosis. Faeces and blood samples were collected during two periods at least 6 months apart. For the healthy animals, both tests were consistently negative over a two-year period, including the one-year period covered by the study. Diagnosis was established as previously described [11].

### DNA extraction, GBS library preparation and sequencing

Genomic DNA was extracted from blood samples using the Wizard Genomic DNA Purification Kit (Promega, Madison, WI) according to the manufacturer’s instructions. The DNA was quantified by spectrophotometry using a NanoDrop ND-1000 spectrophotometer (Thermo Scientific, Wilmington, DE). Libraries were prepared by the Plateforme d’Analyses Génomiques at the Institut de biologie intégrative et des systèmes (Université Laval, Quebec City, QC, Canada). Briefly, two sets of 48-plex GBS libraries were prepared as described in Poland et al. [4] by digesting the genomic DNA with *Pst*I and *Msp*I and ligating each library to its respective barcoded adapters with a *Pst*I overhang. To determine whether complexity reduction could be achieved by selective amplification, a set of 48 samples (24 MAP-infected cows and 24 healthy cows) was amplified by selective primers, as described in Sonah et al. [9], and the same samples were amplified using generic primers. Single-end sequencing (100-bp) was performed on an Illumina HiSeq 2000 by the McGill University and Génome Québec Innovation Centre (Montreal, QC, Canada; http://gqinnovationcenter.com/index.aspx).

### Sequence analysis, alignments and SNP calling

DNA sequence data (100-nt fastq files) were processed using the IGST-GBS pipeline as described in Sonah et al. [9]. Briefly, reads were associated with individual samples by the recognition of the exact sequence of the barcode (4 to 8 bp) followed by TGCAG, the nucleotides that remain after digestions with *Pst*I (FASTX-Toolkit). The sequences were afterwards trimmed to 64 bp and aligned to the reference genome using the Burrows-Wheeler Aligner [12]. The variants were called with PLATYPUS [13] if they had a minimum read depth of 2 and a minimum genotype quality score of 5.

### Analysis of fragments digested by restriction enzymes

#### *In silico* digestion

*In silico* digestion of the bovine chromosome was performed with the *Bos taurus* reference sequence (UMD_3.1.1/BosTau8). The location of fragments produced for each digestion was computed using the program RESTRICT from the EMBOSS version 6.5.7.0 package [14]. Digested fragments for each size range were summed, and their proportion relative to the total number of fragments was reported on the histogram. Relative frequencies of fragment sizes were evaluated by using bin widths of 100 bp.

Estimation of the number of fragments produced through the *Pst*I/*Msp*I GBS assay. To estimate the number of fragments produced through the GBS experiment and to compare them with the *in silico* predictions, the 48 alignments files derived from the conventional method of library preparation were analyzed using GENOMECOV included in the BEDtools. Using the output files from this command and a custom Perl script we computed the sum of predicted fragments for all chromosomes by assuming that all fragments are separated by more or less long spaces where the coverage is 0. We next estimated the average number of *Pst*I/*Msp*I fragments per sample and calculated the standard deviations.

### Bovine SNP50 genotyping and analysis

Individuals sequenced in our GBS assays were also genotyped with the Illumina BovineSNP50 BeadChip array (Zoetis, Kalamazoo, MI). Starting from the Illumina ‘A/B’ allele genotypes, a VCF file containing 67,740 SNPs was produced using a custom Perl script. This script removed duplicate SNPs, SNPs that were not consistent with the nomenclature of ‘Allele A’ and ‘Allele B’ according to the Illumina genotyping system, and SNPs that did not align perfectly (BLASTN) to the reference sequences of the bovine autosomes and mitochondrial genome (UMD_3.1.1/BosTau8) or the X chromosome (Btau4.6.1). After monomorphic SNPs had been removed, a SNP50 dataset containing 50,539 informative SNPs was produced and used for assessing the accuracy of GBS genotypes by direct comparison of the common genotypes.

### Assessment of the accuracy of GBS genotypes produced by the two methods of library preparation

Prior to any filtration of GBS data, common variants shared between the GBS and the SNP50 datasets were used to assess the accuracy of the genotypes derived from the two library preparation methods. We assume that the SNP50 array reported true genotypes. The accuracy of the GBS genotypes was defined as: (the number of identical genotypes between GBS and SNP50/total number of genotypes) * 100. For example, to evaluate the accuracy of the genotypes produced by the conventional-primer approach, the GBS genotypes from 1604 markers from a total of 48 animals were compared directly to their corresponding genotypes in the SNP50 dataset. Missing genotypes were excluded from the computation performed using a custom Perl script. The accuracy of GBS genotypes produced by the two methods was also tested by mass spectrometry. A total of 13 SNPs were genotyped using the Sequenom MassARRAY iPLEX Platform at the McGill University and Génome Québec Innovation Centre. Direct comparisons of the corresponding genotypes were performed, assuming that mass spectrometry reported true genotypes.

### Variant QC filtering

#### First step

The VCF file containing the GBS raw variants produced by the conventional method of library preparation was imported in the Golden Helix SVS software (Golden Helix, Bozeman, MT). To estimate the accuracy of the GBS genotypes selected after filtering under various quality control (QC) conditions, we derived eight datasets from the original dataset using four levels of minimum RD values (≥3, 4, 5 or 6) with or without additional filtering for genotype quality (GQ) ≥ 20 (Table 3). GQ, defined as a conditional genotype quality is encoded as a phred quality score and is equal to -10log_10_ p, where p is the probability that the genotype call is wrong at the site showing a variation. The accuracy of the GBS genotypes has been estimated as described previously by using the markers shared with the BovineSNP50 BeadChip array in each dataset.

#### Second step

For a specific variant, the call rate is the ratio of individuals with known genotypes over the total number of individuals. Considering all genotyped loci, the average call rate can be view as the average proportion of samples per loci for which genotype information is available. With the conventional GBS approach, the average call rate of the raw variants is low, meaning that for most variants, the actual genotypes are missing for a majority of samples. Thus selecting variants with relatively high call rate values would lead to the exclusion of almost all variants from downstream analysis. For variant filtering, the minimum call-rate is the minimum proportion of samples with known genotypes a variant must have in order to be retained. Starting with three datasets that had been previously filtered for minimum read depth and high GQ values (IDs 2, 4 and 6 in Table 3), three low thresholds for minimum call rate (0.2, 0.3, and 0.4) were tested to keep a substantial fraction of the raw variants generated by the IGST-GBS pipeline. Using these thresholds a specific variant was retained if the genotype was found in at least 10, 15, or 19 individuals, respectively, out of 48. At this step, we also tested two values for the minimum minor allele frequency (MAF), 0.02 and 0.05, and eliminated variants if they had two or more alternative alleles.

### Imputation of GBS genotypes

The presence of large proportions of missing data in GBS datasets requires an imputation step to fill these gaps before any association tests can be performed. To evaluate how filtration criteria would influence imputation accuracy, we derived 12 datasets from the original catalogue of conventional-primer GBS variants (see Table 4). We tested different parameters: i) the minimum read depth, ii) the minimum minor allele frequency, iii) the minimum call rate, iv) the imputation program and v) the effect of combining GBS and SNP50 markers to obtain high marker-density conditions before imputation. Two software packages, BEAGLE v4.0 [15] and FIMPUTE v2.2 [16], were used for the imputation of the missing data in the GBS datasets.

We were also interested in evaluating the impact of high marker density on the accuracy of imputed GBS markers. To obtain high marker-density conditions, we firstly removed from the SNP50 dataset all markers shared with the GBS, reducing the number of SNP50 markers from 50,539 to 49,084. Using VCFtools, 12 mixed datasets (GBS + SNP50 in Table 4) were then produced by combining GBS markers obtained with different QC conditions with this unique altered SNP50 dataset. This procedure allows estimating the accuracy of imputed GBS genotypes (and not SNP50 array genotypes) by direct comparison of GBS genotypes shared with those present in the unaltered SNP50 dataset. Before running the imputation programs, monomorphic SNPs, multiple nucleotide variation as well as variants located on chromosome X, Y, or on the mitochondrial genome were removed using the VCFtools. In all cases, the accuracy of imputation of missing GBS data was estimated by comparing the imputed GBS genotypes with those reported by the SNP50 array at all common loci, assuming again that the genotypes produced by the SNP50 array represented true genotypes.

### Data deposition

Variants were deposited in the NCBI dbSNP database under the batch ID GSB_18_48 for the conventional-primer dataset and under the batch ID GSB_19_48 for the selective-primer dataset.

## Results

### *In silico* restriction enzyme digestions to confirm suitability of *Pst*I/*Msp*I for sequencing bovine GBS libraries

**Fig. 1:**
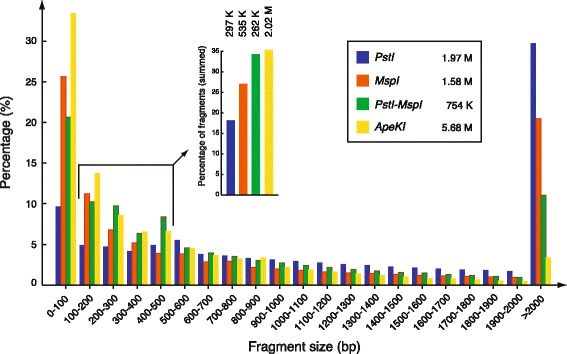
*In silico* analysis of restriction enzyme sites in the bovine genome. The percentage was calculated based on the number of fragments obtained with the respective digestion that fall within each range of fragment lengths over the total number of fragments obtained with the corresponding restriction enzyme digestion. The total number of fragments obtained with the corresponding restriction enzyme is indicated in the legend box. The number of fragments computed in the size range between 100 and 500 bp is indicated above the corresponding bar of the small histogram

Digestion of the 2.6-billion-bp bovine genome with a 6-bp restriction enzyme has the potential to generate more than 634,000 fragments, most of which are greater than 1 kbp, a size that is too large to be efficiently sequenced by Illumina’s HiSeq systems. To reduce the number of fragments produced and to maximise the proportion of fragments with optimal sizes for sequencing (100 to 500 bp), the two-enzyme version of the GBS protocol proposed by Poland et al. [4] uses a combination of a ‘medium frequency cutter’, *Pst*I (CTGCAG), with a ‘frequent cutter’, *Msp*I (CCGG). To make sure that these two enzymes were suitable for the bovine genome, we performed an *in silico* digestion of the bovine chromosome (Fig. 1) and compared the predicted fragment-size distribution with other enzymes used in recent GBS studies. As shown in Fig. 1, *Ape*KI and the combination of *Pst*I and *Msp*I produced the larger proportions of fragments in the size range between 100 and 500 bp relative to the other enzymes. Digestions of the bovine genome with *Pst*I/*Msp*I generated a total of 754,306 fragments, fewer than those produced by single-enzyme digestions with *Ape*KI (5.68 million), *Pst*I (1.58 million), or *Msp*I (1.97 million). We therefore concluded that the combination of *Pst*I and *Msp*I has the potential to significantly reduce the complexity of the bovine genome, because these enzymes target fewer sites than single digestions with *Pst*I, *Msp*I or *Ape*KI do, a situation that may increase the sequence coverage of the resulting fragments.

**Fig. 2:**
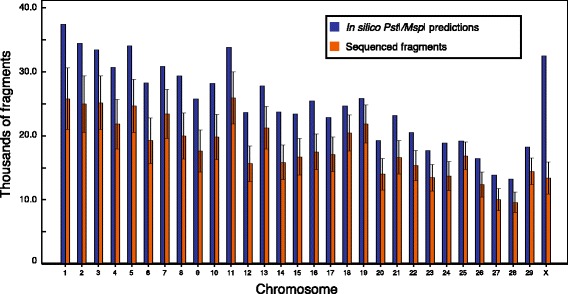
Comparison of the average number of sequenced *Pst*I/*Msp*I fragments with the corresponding *in silico* predictions. The average numbers of fragments sequenced per animal as well as the standard deviations were deduced from the alignments files

We have also evaluated the distribution of fragments sequenced through our *Pst*I/*Msp*I assay to compare them with the *in silico* predictions. We found that for all autosomes, the average numbers of fragments produced per animal are slightly lower than those predicted *in silico* (Fig. 2).

### Comparison of a conventional two-enzyme GBS protocol and a modified method with selective primers

**Fig. 3:**
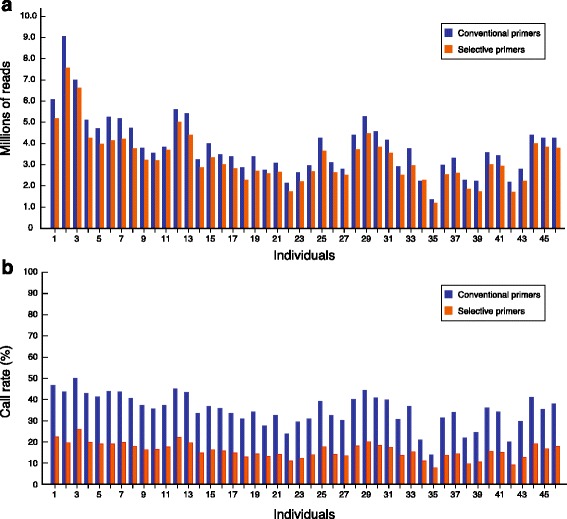
Distribution of the number of reads and variant call rates. **a** Number of mapped reads assigned to individual samples after demultiplexing of two 48 plex sequencing lanes. One group of libraries was prepared with conventional primers, and the other with selective primers. **b** Call rate of the variants in both methods of library preparation. Variants detected have a minimum read depth of 2 and a genotype quality score of 5 or greater

Single-end sequencing with the conventional method of library preparation produced a total of 191,912,978 reads, with an average of 4.0 million reads per sample (Table 1). The GBS method with selective primers generated a total of 163,583,652 reads, with an average of 3.4 million reads per sample (Table 1). The number of raw sequence reads per individual ranged from 1.4 million to 9.1 million with the conventional method and from 1.2 million to 7.6 million with selective primers (Fig. 3a). Of the 48 samples, two individuals failed the bovine SNP50 genotyping assay and were removed from further comparison analysis.

**Table 1:** Descriptive features of GBS data generated with two methods of library construction

	Total	Library construction	
		Conventional primers	Selective primers
Raw reads		191,912,978	163,583,652
Mapped reads		186,122,054	159,145,610
GBS variants		272,103	123,666
	Average call rate (*n* = 46)	0.352 [0.140–0.501]	0.159 [0.078–0.260]
	Common with SNP50	1604	756

A total of 272,103 variants were detected with the conventional GBS approach, whereas 123,666 variants were found with the selective-primer approach (Table 1). It should be noted that the yield of the sequenced lanes (192 million versus 186 million) cannot explain this difference. The lower number of variants associated with the latter method was largely expected, since it was designed to select a subset of the digested fragments in order to increase the sequence coverage associated with genotypes. As expected, we found a high proportion of individuals with missing genotypes in both datasets. In the panel of variants derived from the conventional-primer approach, the call rate per sample ranged from 0.140 to 0.501, for an average value of 0.352 (Table 1, Fig. 3b). Lower call rates were recorded with the selective-primer approach, with values ranging from 0.0784 to 0.260.

**Fig. 4:**
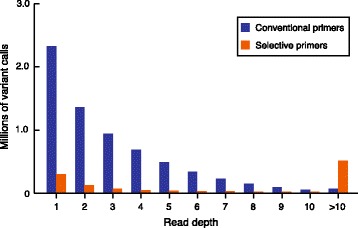
Distribution of read-depth coverage in genotype calls for both methods of library preparation. The conventional-primer method outperformed the method with selective primers on the basis of the number of genotype calls reported. The selective-primer method produced more calls with read-depth values greater than 10, whereas the conventional-primer method produced more genotypes with 10 reads or fewer

By looking at the raw data generated by the pipeline, we found that a large proportion of the variants were supported by low read-depth values (RD < 10). We further examined the distribution of all variant calls according to their associated read-depth coverage in both methods (Fig. 4). At low RD values (<10), the conventional-primer method produced more variants calls than the selective-primer method did, in contrast to the situation observed at high read-depth values, where more calls were generated by the selective-primer method. This result confirms that the latter library preparation method is very effective for increasing the read-depth coverage at the expense of the number of sites that could be genotyped. Overall, the selective-primer method produced fewer genotype calls than the conventional-primer method did, and a large proportion of those genotypes had a much higher read depth.

### Assessing the accuracy of GBS genotypes

To assess the accuracy of the genotypes reported by the two methods, 13 markers under investigation for their association with Johne’s disease (not shown) were genotyped using the Sequenom MassARRAY iPLEX Platform. As shown in Table 2, all GBS genotypes derived from the conventional-primer method were concordant with genotypes reported by the MassARRAY technique, suggesting that the conventional-primer protocol produced genotypes with high accuracy. In contrast, a substantial portion of the selective-primer genotypes did not agree with those reported by the Sequenom platform (Table 2). In all but one discordant case, true heterozygotes were miscalled as homozygotes by the selective-primer approach.

**Table 2:** GBS calls relative to Sequenom MassARRAY genotypes^a^

Conventional-primer method										
				Concordant			Discordant			Concordance (%)^b^
Chr	Position	Ref	Alt	MM^b^	Mm^b^	mm^b^	MM	Mm	mm	
5	124968560	C	T	0	11	5	0	0	0	
6	120556623	T	C	1	8	0	0	0	0	
7	25377202	A	G	0	2	1	0	0	0	
12	81073590	G	T	5	4	0	0	0	0	
17	4536929	G	A	2	8	0	0	0	0	
18	11257808	A	G	2	8	0	0	0	0	
18	63490716	C	T	6	17	0	0	0	0	
23	18485367	C	G	2	8	0	0	0	0	
28	1121069	G	T	5	5	0	0	0	0	
		Total		23	71	6	0	0	0	100.0
Selective-primer method										
5	124968560	C	T	3	13	16	0	9	0	
6	120556623	T	C	10	22	12	0	2	0	
7	25377202	A	G	5	17	19	0	1	1	
11	6123151	T	C	18	13	3	0	8	0	
12	81073590	G	T	19	11	3	0	7	0	
17	4536929	G	A	11	11	12	0	9	0	
18	11257808	A	G	20	16	6	0	3	0	
18	63490716	C	T	17	13	4	0	7	0	
23	18485367	C	G	18	13	2	0	4	0	
24	26394985	G	T	18	13	7	0	5	0	
24	26395035	C	T	7	14	18	0	6	0	
28	1121069	G	T	24	9	0	0	6	0	
X	49928152	C	T	34	8	0	0	0	0	
		Total		204	173	102	0	68	0	87.6

^a^The 13 single-nucleotide polymorphisms (SNPs) examined were initially detected in a selective-primer dataset. Only 9 of these 13 SNPs could be retrieved in the corresponding conventional-primer dataset. Variants in these datasets were kept if they qualified based on a read depth ≥ 5, a genotype quality score ≥ 20, no more than one alternative allele, a call rate > 0.2, and a minor allele frequency ≥ 0.025. Chr: chromosome; Ref: reference allele; Alt: alternate allele; MM: homozygous for the reference allele; Mm: heterozygous; mm: homozygous for the alternate allele

^b^The reliability of SNP prediction was based on the reference calls predicted using the Sequenom MassARRAY technology. Discordant SNP reports call conflict by GBS

We also examined the variants shared between the SNP50 array and the two library preparation approaches. The 50,539 unique SNPs retained in the SNP50 dataset were largely independent from those obtained with the GBS approach, with only 1604 markers common to the conventional-primer and SNP50 datasets and 756 markers common to the selective-primer and SNP50 dataset (Table 1). Although small, the marker overlap between the GBS and SNP50 array datasets allowed us to compare directly the accuracy of the genotypes produced by the two methods. In the absence of any filtration step, we found that the accuracy of the genotypes produced by the selective-primer approach is around 69.7% whereas that of the genotypes derived from the conventional approach reach 89.0%. This result confirmed our initial finding that a significant proportion of the GBS genotypes produced by the latter approach were miscalled by this method.

**Fig. 5:**
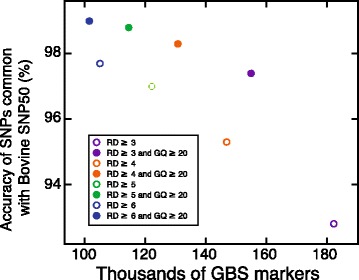
Estimated accuracies of GBS genotypes after the first quality control step. Accuracy was estimated using GBS variants that were also found on the SNP50 array. The GBS genotypes were filtered according to their associated read-depth (RD) coverage without consideration of the genotype quality (GQ) or using a GQ score threshold of 20. At each shared locus, the concordance of the GBS and the SNP50 genotypes was assessed, assuming that those reported by the SNP50 array represented true genotypes

Working henceforth with GBS genotypes produced by the conventional-primer approach, we were initially interested in determining the minimum number of reads required to call a genotype with confidence. We also examined how filtering simultaneously for GQ could affect the accuracy of genotypes. As shown in Fig. 5, we found that the minimum number of reads required to call a genotype had a great impact on the number of variants that passed this first filtering step. Again, this is explained by the above-mentioned distribution of read-depth values associated with genotypes (Fig. 4). Interestingly, when datasets were not filtered according to GQ, the accuracy substantially decreased, but this gap was reduced when the minimum read-depth threshold was increased (Fig. 5). For example, genotypes in dataset 1 (Table 3) had an overall estimated accuracy of only 92.8% in comparison with those in dataset 7 (97.7%). To explain this result, one must realise that many variants included in this dataset were called using only three reads and that the probability of miscalling a heterozygote using three reads is high, at 1 in 4. In contrast, simultaneous filtering for GQ consistently increases the genotype accuracy, because genotype calls having a GQ score greater than 20 (encoded as a Phred score) have a greater than 99% probability of being real.

**Table 3:** Effect of read-depth and genotype quality (GQ) thresholds on the accuracy of GBS genotype calls

Dataset ID	Filtering criteria (first step)	Number of variants	Markers shared with SNP50	Number of calls examined	Estimated accuracy (%)^a^
1	≥3 reads	182,334	1150	20,845	92.8
2	≥3 reads and GQ > 20	154,991	1006	11,029	97.4
3	≥4 reads	146,936	956	14,552	95.3
4	≥4 reads and GQ > 20	130,813	865	8873	98.3
5	≥5 reads	122,234	825	9818	97.0
6	≥5 reads and GQ > 20	114,588	778	6952	98.8
7	≥6 reads	105,085	723	6478	97.7
8	≥6 reads and GQ > 20	101,552	689	5147	99.0

^a^The reliability of GBS genotypes was based on the reference calls generated using the SNP50 BeadChip. Estimation of the concordant SNPs is reported

### Importance of adjusting the minimum call-rate threshold in the second step of QC filtering

**Fig. 6:**
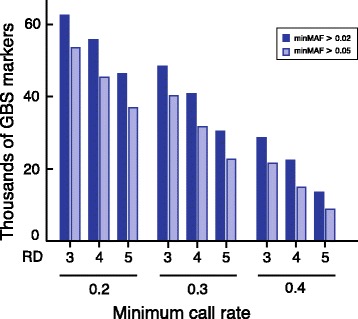
Effect of different quality control parameters on the number of GBS markers selected for downstream analysis. The markers identified by GBS and used in the 18 datasets had a genotype quality score ≥ 20 and no more than one alternative allele. More details on the datasets can be found in Table 3. minMAF: minimum minor allele frequency; RD: read depth

As shown in Fig. 6, the number of remaining markers after the second QC step decreases linearly with an increase in the minimum call rate. For example, when using a minimum of three reads to call a genotype, the number of markers retained decreases from 56,556 markers at a call rate of 0.2 or greater to 21,561 markers at a call rate of 0.4 or greater (Table 4). As expected, we found that decreasing the minimum MAF threshold from 0.05 to 0.02 allows slightly more variants to be retained. The results presented in Fig. 6 clearly highlight the importance of considering a less-stringent minimum call rate to make sure that enough variants are retained for downstream analysis.

**Table 4:** Description of datasets used for testing different parameters on the accuracy of imputation of GBS missing data

Dataset ID	Description	Min. read depth	MinMAF^a^	Min. call rate	Number of variants	Variants shared with SNP50	Imputation program	Estimated accuracy (%)
9	GBS	3	0.05	0.2	53,556	431	FImpute	71.1
10	GBS + SNP50	3	0.05	0.2	98,398	431	FImpute	84.8
11	GBS	3	0.05	0.3	40,208	334	FImpute	74.2
12	GBS + SNP50	3	0.05	0.3	88,573	334	FImpute	89.1
13	GBS	3	0.05	0.4	21,561	156	FImpute	77.5
14	GBS + SNP50	3	0.05	0.4	70,250	156	FImpute	91.9
15	GBS	4	0.02	0.2	55,727	395	FImpute	73.3
16	GBS + SNP50	4	0.02	0.2	100,297	395	FImpute	86.4
17	GBS	4	0.05	0.2	45,447	369	Beagle	70.9
	GBS	4	0.05	0.2	45,447	369	FImpute	71.2
18	GBS + SNP50	4	0.05	0.2	90.234	369	Beagle	78.5
	GBS + SNP50	4	0.05	0.2	90.234	369	FImpute	86.1
19	GBS	4	0.02	0.3	40,855	278	FImpute	75.9
20	GBS + SNP50	4	0.02	0.3	89,196	278	FImpute	90.3
21	GBS	4	0.05	0.3	31,662	254	Beagle	74.0
	GBS	4	0.05	0.3	31,662	254	FImpute	74.3
22	GBS + SNP50	4	0.05	0.3	80,190	254	Beagle	83.8
	GBS + SNP50	4	0.05	0.3	80,190	254	FImpute	89.3
23	GBS	4	0.02	0.4	22,331	98	FImpute	83.3
24	GBS + SNP50	4	0.02	0.4	70,983	98	FImpute	93.4
25	GBS	4	0.05	0.4	14,909	82	Beagle	81.2
	GBS	4	0.05	0.4	14,909	82	FImpute	80.8
26	GBS + SNP50	4	0.05	0.4	63,706	82	Beagle	89.8
	GBS + SNP50	4	0.05	0.4	63,706	82	FImpute	93.4
27	GBS	5	0.05	0.2	36,913	299	FImpute	70.0
28	GBS + SNP50	5	0.05	0.2	81,454	299	FImpute	85.4
29	GBS	5	0.05	0.3	22,685	166	FImpute	74.8
30	GBS + SNP50	5	0.05	0.3	71,360	166	FImpute	89.5
31	GBS	5	0.05	0.4	8818	42	FImpute	79.5
32	GBS + SNP50	5	0.05	0.4	57,719	42	FImpute	92.2

^a^MinMAF: minimum minor allele frequency

### Imputation accuracy of missing data

**Fig. 7:**
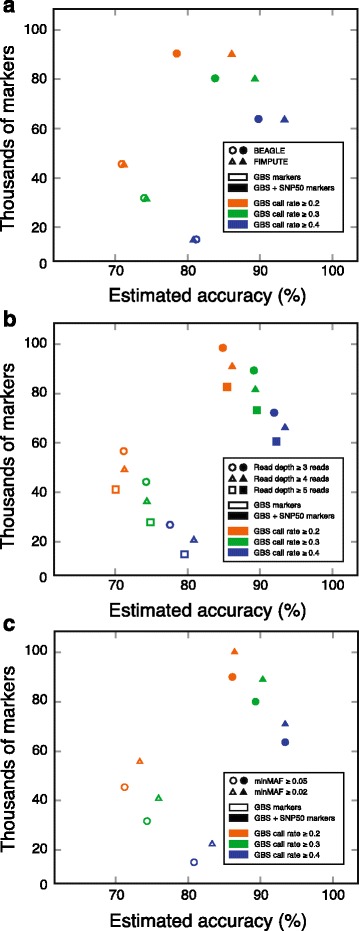
Effect of different parameters on the accuracy of imputation of GBS markers and the number of variants retained. The accuracies of the missing GBS genotypes were estimated by evaluating the concordance of the imputed GBS genotypes with the SNP50 genotypes. All panels illustrate the effect of the minimum call rate as well as the combination of GBS markers (genotype quality score ≥ 20) and SNP50 markers on the estimated accuracy of GBS-imputed markers. The parameters examined were **a** the imputation program, **b** the thresholds for the minimum read depth, and **c** the minMAF

Two programs, BEAGLE and FIMPUTE, were used for the imputation of the missing data. BEAGLE is an established genetic analysis software program that uses hidden Markov models, exploits linkage disequilibrium (LD) between markers (population imputation method) and assumes that all animals are unrelated [17]. On the other hand, FIMPUTE is a deterministic software designed to handle large-scale genotype imputation in livestock [16]. We noticed that both programs performed equally well when imputing datasets containing GBS markers only, whereas FIMPUTE clearly outperformed BEAGLE when SNP50 and GBS markers were combined (Fig. 7a). In addition, the most striking result presented in Fig. 7 was the improved imputation accuracy of the missing GBS genotypes observed in datasets combining GBS and SNP50 genotypes. The imputation accuracy also consistently increased when higher minimum call-rate thresholds were applied, with the highest accuracies obtained with a call rate of 0.4 or greater (Fig. 7). However, GBS datasets filtered with this threshold contain many fewer markers than datasets filtered with lower call rate thresholds do.

We next asked how changes in the threshold for the minimum read depth associated with genotypes could affect the accuracy of imputation. Our results indicate that the minimum read-depth threshold hardly impacts the estimated accuracy of the imputed genotypes (Fig. 7b). This finding is not surprising, given that we already showed that the estimated accuracies of genotypes increased only moderately when the minimum read-depth threshold was raised from three reads to four or five, provided that genotypes were also filtered with GQ scores of 20 or greater (Fig. 5). In light of these observations (Fig. 7b), one can conclude that the gains in genotype accuracy of using four or five reads relative to only three reads are marginal and do not offset the cost of losing thousands of markers.

The effect of lowering the threshold for the minimum MAF on the estimated accuracy of imputed genotypes is presented in Fig. 7c. We found that datasets consisting of variants with a MAF greater than 0.02 were slightly larger and better imputed than their corresponding datasets with a MAF greater than 0.05 were. This observation is surprising, given that rare alleles were generally found to be harder to impute than their more widespread counterparts [16, 18].

## Discussion

### GBS for SNP discovery and genotyping in a cattle population

In the present study, we developed strategies to deal with two major issues of the GBS technique when used as a genotyping assay: its low-coverage sequencing and the presence of high levels of missing data. To our knowledge, this study is the first to examine how the read-depth coverage and other QC parameters affect the accuracies of the genotypes produced by this method. We used GBS as a technique to genotype markers in a bovine population with relatively high heterozygosity rates, in contrast to the situation in many GBS crop studies, where selection resulted in decreased heterozygosity rates. In doubled haploids, genotypes can be called accurately with one or two reads, because almost all genotypes of an individual are homozygotes for the major or minor alleles. In contrast, using GBS to genotype animal populations poses a major challenge, because a minimum number of reads are needed to call a heterozygote with confidence.

From a SNP discovery perspective, our report allowed the detection of 272,103 variants using the conventional-primer method, more than the 63,697 SNPs previously identified in a similar GBS study in cattle [5]. Given that the two studies used a similar sample size and were performed with the same multiplexing level, we believe that the increased number of variants detected in our study is the result of our decision to use a two-enzyme protocol rather than a single digestion with *Pst*I. In both studies, sequencing reactions are anchored on the *Pst*I site. However, in our study, the addition of the ‘common cutter’ *Msp*I increased the number of fragments available for sequencing by cutting the large *Pst*I-*Pst*I fragments that otherwise could not have been sequenced. A two-enzyme protocol is therefore an elegant strategy to improve the number of sampled sites. The distribution of the sequenced fragments compared with the in silico *Pst*I/*Msp*I prediction (Fig. 2) suggests a great performance of the conventional method of library preparation. The reduced number of sequenced fragments relative to the *in silico* prediction is likely explained by a significant proportion of the *Pst*I or *Msp*I restriction sites that are potentially methylated in the genome. Nevertheless, we expect that a larger case–control cohort should generate more SNPs because it will capture a larger proportion of the genetic variability present in the population. For example, a recent study using the *Pst*I GBS protocol for SNP identification reported 515,787 SNPs among 1246 Canadian Holstein cows [6]. Consequently, by using such a large cohort of dairy cattle for the association with Johne’s disease, we expect to identify a much larger number of SNPs. Because many genotypes in our study are covered by only 1 or 2 reads (Fig. 4), it is clear that a slight decrease in the multiplexing level could also increase the number of variants retained after the QC filtering steps.

The catalogue of variants detected by De Donato et al. [5] displayed a much higher call rate (88.1%) than what we observed in our variant dataset (35%; Table 1, Fig. 3b). Similar high call rates (>85%) were also reported in a second study using GBS to genotype dairy cows [6]. Although the nature of the informatics pipelines and the sequencing output could be evoked to explain these differences, it is clear that most of them are attributable to the fact that these studies reported genotypes supported by only one read. In contrast, we reported genotypes when two or more reads were present at the same locus through the IGST-GBS pipeline, with the consequence that more missing data were included in our catalogue of raw variants (Fig. 3b).

Our decision to use a minimum of three reads was based on the consideration that minimum coverage should be used to call a heterozygous animal. There is no way that a heterozygous individual could be adequately called with one read. Our pipeline includes an efficient SNP calling tool (PLATYPUS) that retains variants supported by at least two reads per locus. We considered two reads to be inadequate, with only a 50% chance of accurately calling a heterozygous animal. Adequate coverage of a heterozygote requires that both chromosomes of a diploid individual have a reasonable chance of being sequenced at a specific site, and such odds are generally not possible when markers are derived from low-coverage sequencing techniques (<5X per site per individual) [19]. Nevertheless, using our QC parameters, which included a GQ score of 20 or greater and a read depth of 3 or greater, we selected variants that had more than 97% accuracy (Fig. 5). Missing data for these highly qualified markers were then imputed with an estimated accuracy greater than 85% (Fig. 7). In light of these observations, one can conclude that the gains in genotype accuracy of using four or five reads relative to only three reads are marginal and do not offset the cost of losing thousands of markers (Fig. 6). We are confident that the information provided by genotypes imputed with such accuracies could be useful for detecting variants associated with Johne’s disease. For example, Ibeagha-Awemu et al. [6] retained GBS markers imputed with an accuracy greater than 50% in their GWAS that identified novel candidate genes influencing cow milk traits.

De Donato et al. [5] removed from their analysis SNPs present in less than 70% of the population as well as individuals with low read numbers. We choose to not perform such sample QC filtering, but it is clear from the distributions of reads and call rates presented in Fig. 3 that removing individuals with low read numbers could be beneficial in terms of the number of variants retained after filtering for call rate. Such sequencing bias between samples was observed in other GBS studies and is thought to originate mainly from variations in DNA quality between samples [5, 20].

To preserve the low cost per sample that the GBS method offers, we examined how QC criteria combined with different parameters of filtration impact genotype accuracy and the number of variants retained. We found that high number of accurate genotypes could be selected using low values (3 to 5) for the minimum read-depth thresholds, provided that genotypes are simultaneously filtered according to their GQ scores. We also tested a modified GBS approach that uses selective primers for amplifying a smaller fraction of the DNA cut with the restriction enzymes. We found that a sizeable proportion of true heterozygote genotypes were miscalled by this method. Interestingly, at most of the loci examined, we observed that true heterozygotes were called correctly in certain individuals and miscalled as homozygotes for the major or minor alleles in other individuals (data not shown), making it difficult to pinpoint the source of the problem that occurred in library preparation. Nevertheless, we hypothesise that the addition of the two nucleotides at the end of the 3′ primers may cause problems in the annealing step in some circumstances and could prevent the adequate amplification of one allele.

### Accuracy of imputation of missing data

Given the preponderance of missing data in GBS datasets, testing the accuracy of imputation of missing data is of utmost importance. We used shared variants between GBS and SNP50 datasets to assess the accuracy of imputed GBS genotypes under different conditions. Using two imputation software programs, we found that FIMPUTE gave consistently higher imputation accuracies than BEAGLE did when enhanced datasets (GBS + BovineSNP50) were considered. FIMPUTE is not only more accurate than BEAGLE is in cattle datasets but also requires considerably less computer resources, an advantage when working with large population sizes. We chose to not use the pedigree information in our imputation analyses because of the limited number of samples included in this study. With a larger cohort, it would be interesting to evaluate whether this information benefits the quality of imputation. A recent study in *Bos indicus* dairy cattle showed that imputation scenarios using pedigree information and FIMPUTE resulted in similar or slightly lower accuracies in comparison to scenarios in which pedigree information was not used [18]. In our study, the FIMPUTE-estimated accuracy of imputation of GBS data ranged from 84.8% to 93.4% for combined datasets, similar to the accuracy of 93% to 95% observed in soybean datasets that tolerate up to 80% missing data [10]. The slightly higher imputation accuracy observed in soybean lines was likely due to the fact that highly homozygous lines with a residual percentage of heterozygosity around 3% were used (D. Torkamaneh, personal communication), a situation that may facilitate the task of imputation programs, because we assume that most imputation errors imply heterozygote genotypes. Conversely, the high heterozygosis rate observed in the Holstein population (34.2% in our study) could make the task of accurately imputing genotypes harder.

Our data indicate that combining markers from the SNP50 array dataset and the GBS dataset increased the accuracy of imputation of missing GBS genotypes. This finding is supported by earlier reports that the accuracy of imputation is dependent on the density of markers [18, 21, 22]. Contrary to the aforementioned observations in soybean [10], we did not find higher imputation accuracies in datasets containing a larger proportion of missing data (up to 80%). Rather, our results indicate that benefits for the accuracy of imputation of GBS genotypes result from increases in the minimum call rate (Fig. 7). Based on this finding, we expect that in future studies, decreasing the level of multiplexing or removing individuals with low call rates will positively affect the accuracy of imputation. According to Torkamaneh and Belzile [10], increasing the portion of missing data tolerated in GBS datasets also increased the imputation accuracy of missing data, because additional markers helped better capture the haplotype diversity present in the population. The lower imputation accuracies observed in our datasets with more missing data (those with the lower minimum call rates) could possibly be explained by the size of the cattle haplotypes, which are much shorter than their counterparts in the soybean genome. Previous reports in cattle populations have shown that the decline in LD as a function of distance is rapid, with average LD (*r* ^2^) dropping below 0.2 at distances of 50 kb [23] and below 0.1 at 100 kb [24]. We can hypothesise that most markers added by lowering the minimum call rate will have low LD values and may help the imputation of flanking markers only a little. The impact on the overall estimated accuracy could be negative, because these markers, which contain a higher proportion of missing data, may be harder to impute accurately. However, we cannot rule out that other factors, notably the marked differences in the heterozygosity rate, may have played a role in these contrasting results.

## Conclusions

The results presented here are promising in many respects: the conventional two-enzyme GBS protocol was proved to be useful for producing accurate genotypes at a low cost per sample. Using low thresholds for call rate and read-depth coverage, combined with simultaneous filtering for GQ values, we were able to select 53,556 GBS markers from only 48 animals without sacrificing the quality of the genotypes. In addition, the accuracy of imputation of missing data was proved to be highly accurate in spite of the limited number of samples included in our analysis. In our future work, the GBS technique detailed here will be used to genotype a larger cohort of animals (>1000 individuals) with different MAP infection statuses. To maintain a low cost per sample, it is likely that the GBS data obtained will be used to impute the markers present on the SNP50 array. Using complementary datasets and the strategies detailed here, we are confident that we could pilot an original GWAS with an unrivalled number of markers in comparison with the most recent association studies that have explored susceptibility to MAP infection in cattle [22]. In our opinion, the proposed variant QC filtering and validation performed in this study qualified the *Pst*I/*Msp*I GBS assay as a low-cost high-density genotyping platform that presents many advantages over the array-based genotyping platforms. Our results could therefore have practical implications in the field of applied cattle breeding, notably in paving the way to the development of Genomic selection models using GBS markers. From a broader perspective, the conclusions reported here regarding RD and GQ filtering could benefit many GBS applications, notably GWAS, but also QTL mapping, genomic selection, phylogeography or population genomics. In livestock, there is a need to increase the density of markers genotyped across the target population while preserving the quality of genotypes. However, one must keep in mind that low-coverage genotyping methods are realistic only in species with an available reference genome and for which there is a possibility to impute missing genotypes using a reference panel.

## Abbreviations

GBS: Genotyping-by-sequencing
GQ: Genotype quality
GWAS: Genome-wide association study
LD: Linkage disequilibrium
MAF: Minor allele frequency
MAP: Mycobacterium avium *subsp*. paratuberculosis
PCR: Polymerase chain reaction
QC: Quality control
RD: Read depth
SNP: Single-nucleotide polymorphism

## References

[1] Davey JW, Hohenlohe PA, Etter PD, Boone JQ, Catchen JM, Blaxter ML (2011). Genome-wide genetic marker discovery and genotyping using next-generation sequencing. Nat Rev Genet.

[2] Elshire RJ, Glaubitz JC, Sun Q, Poland JA, Kawamoto K, Buckler ES, Mitchell SE (2011). A robust, simple genotyping-by-sequencing (GBS) approach for high diversity species. PLoS One.

[3] Poland JA, Rife TW (2012). Genotyping-by-sequencing for plant breeding and genetics. Plant Genome.

[4] Poland JA, Brown PJ, Sorrells ME, Jannink JL (2012). Development of high-density genetic maps for barley and wheat using a novel two-enzyme genotyping-by-sequencing approach. PLoS One.

[5] De Donato M, Peters SO, Mitchell SE, Hussain T, Imumorin IG (2013). Genotyping-by-sequencing (GBS): a novel, efficient and cost-effective genotyping method for cattle using next-generation sequencing. PLoS One.

[6] Ibeagha-Awemu EM, Peters SO, Akwanji KA, Imumorin IG, Zhao X (2016). High density genome wide genotyping-by-sequencing and association identifies common and low frequency SNPs, and novel candidate genes influencing cow milk traits. Sci Report.

[7] Matukumalli LK, Lawley CT, Schnabel RD, Taylor JF, Allan MF, Heaton MP, O’Connell J, Moore SS, Smith TP, Sonstegard TS (2009). Development and characterization of a high density SNP genotyping assay for cattle. PLoS One.

[8] Stothard P, Choi JW, Basu U, Sumner-Thomson JM, Meng Y, Liao X, Moore SS (2011). Whole genome resequencing of black Angus and Holstein cattle for SNP and CNV discovery. BMC Genomics.

[9] Sonah H, Bastien M, Iquira E, Tardivel A, Legare G, Boyle B, Normandeau E, Laroche J, Larose S, Jean M (2013). An improved genotyping by sequencing (GBS) approach offering increased versatility and efficiency of SNP discovery and genotyping. PLoS One.

[10] Torkamaneh D, Belzile F (2015). Scanning and filling: ultra-dense SNP genotyping combining genotyping-by-sequencing, SNP array and whole-genome resequencing data. PLoS One.

[11] Fock-Chow-Tho D, Topp E, Ibeagha-Awemu EA, Bissonnette N (2017). Comparison of commercial DNA extraction kits and quantitative PCR systems for better sensitivity in detecting the causative agent of paratuberculosis in dairy cow fecal samples. J Dairy Sci.

[12] Li H, Durbin R (2009). Fast and accurate short read alignment with burrows-wheeler transform. Bioinformatics.

[13] Rimmer A, Phan H, Mathieson I, Iqbal Z, Twigg SR, Consortium WGS, Wilkie AO, McVean G, Lunter G (2014). Integrating mapping-, assembly- and haplotype-based approaches for calling variants in clinical sequencing applications. Nat Genet.

[14] Rice P, Longden I, Bleasby A (2000). EMBOSS: the European molecular biology open software suite. Trends Genet.

[15] Browning BL, Browning SR (2016). Genotype imputation with millions of reference samples. Am J Hum Genet.

[16] Sargolzaei M, Chesnais JP, Schenkel FS (2014). A new approach for efficient genotype imputation using information from relatives. BMC Genomics.

[17] Browning SR, Browning BL (2007). Rapid and accurate haplotype phasing and missing-data inference for whole-genome association studies by use of localized haplotype clustering. Am J Hum Genet.

[18] Boison SA, Santos DJ, Utsunomiya AH, Carvalheiro R, Neves HH, O’Brien AM, Garcia JF, Solkner J, da Silva MV (2015). Strategies for single nucleotide polymorphism (SNP) genotyping to enhance genotype imputation in Gyr (Bos indicus) dairy cattle: comparison of commercially available SNP chips. J Dairy Sci.

[19] Nielsen R, Paul JS, Albrechtsen A, Song YS (2011). Genotype and SNP calling from next-generation sequencing data. Nat Rev Genet.

[20] Wong MM, Gujaria-Verma N, Ramsay L, Yuan HY, Caron C, Diapari M, Vandenberg A, Bett KE (2015). Classification and characterization of species within the genus lens using genotyping-by-sequencing (GBS). PLoS One.

[21] Khatkar MS, Moser G, Hayes BJ, Raadsma HW (2012). Strategies and utility of imputed SNP genotypes for genomic analysis in dairy cattle. BMC Genomics.

[22] Zheng J, Li Y, Abecasis GR, Scheet P (2011). A comparison of approaches to account for uncertainty in analysis of imputed genotypes. Genet Epidemiol.

[23] Porto-Neto LR, Kijas JW, Reverter A (2014). The extent of linkage disequilibrium in beef cattle breeds using high-density SNP genotypes. Genet Sel Evol.

[24] Villa-Angulo R, Matukumalli LK, Gill CA, Choi J, Van Tassell CP, Grefenstette JJ (2009). High-resolution haplotype block structure in the cattle genome. BMC Genet.

